# *Morus alba*: natural and valuable effects in weight loss management

**DOI:** 10.3389/fcdhc.2024.1395688

**Published:** 2024-10-25

**Authors:** Foteini Ntalouka, Athina Tsirivakou

**Affiliations:** Department of Research and Development, Herbalist P.C., Athens, Greece

**Keywords:** *Morus alba*, white mulberry, weight loss, diabesity, mulberry’s safety

## Abstract

Overweight and obesity are conditions associated with serious comorbidities, such as diabetes and cardiovascular disease. Prevalence of excessive fat accumulation is increasing worldwide, and thus the need for efficient and sustainable weight loss regimes has become a major issue in clinical practice. Despite the important advances in the development of anti-obesity medications (AOM), their side effects, cost, and accessibility, are limiting factors for their routine use. Conversely, the studies of medicinal plants for weight management holds strong promise as a growing area of research. This review consolidates the representative evidence about the beneficial impacts of *Morus alba* on weight management and associated metabolic parameters, encompassing: inhibition of digestive enzymes, and thus contribution to the energy deficit required for weight loss, improvements in glucose and lipid metabolism, and attenuation of adiposity. Findings from *in vitro, in vivo*, and clinical investigations reviewed in the paper, demonstrate that white mulberry extracts have the potency to supplement efficiently and safely a healthy weight management approach.

## 1 Introduction

Obesity is a multifactorial health condition acknowledged as a worldwide pandemic. According to the World Health Organization, in the latest reports from 2016, 39% of the global population were overweight, whereas 13% were classified as obese. These percentages are notably higher when considering western regions, such as the United States and Europe (1, 2).

Numbers are expected to rise importantly in the next decades. Nevertheless, it is also concerning that populations with traditionally lower prevalence rates, such as the Asian nations, are now experiencing an escalating rate in the prevalence of obesity (3).

The consistently increasing prevalence of overweight and obesity is mainly attributed to the shift from traditional lifestyle and dietary patterns to a more “Western” type of lifestyle, that is characterized by an increased intake of energy-dense foods and a reduction in physical activity. Genetic, environmental, behavioral, and sociocultural influences also play a role in caloric excess that can lead gradually to excessive body fat (4, 5).

Being overweight or obese raises importantly the risk for several life-threatening non-communicable diseases, such as cardiovascular disease, type 2 diabetes, hypertension, and cancer among others. Due to the dramatic impact of obesity in health, it is of utmost importance for the scientific community to identify and endorse the implementation of effective and safe interventions for overweight and obesity management.

The World Health Organization (WHO) uses the body mass index (BMI) to define overweight and obesity in adults. Individuals with a BMI of 25–29.9 are considered overweight, while those with a BMI of 30–39.9 are considered obese. However, such classification does not consider the body mass composition (fat versus muscle mass distribution) and other metabolic features (glucose and lipid metabolism) that may significantly affect overall health. To achieve a “healthy weight” loss, and most importantly maintenance, it is essential to apply a strategy that tackles any metabolic disorders accompanying the excessive body weight and that addresses the needs of the individual, ensuring the critical factor of long-term adherence.

Diet and exercise are strongly recommended as the primary interventions for overweight individuals and can help them lose approximately 3-5% of their weight. In many cases, especially in obesity or in overweight with comorbidities such as diabetes, the combination of lifestyle measures with pharmacotherapy is essential, not only to achieve but also to maintain weight loss. Moreover, multidisciplinary approaches, including behavioral and psychological support can help further patients to improve overall their health status and quality of life (6, 7).

Anti-obesity medications (AOM) development has evolved importantly during the past few years, mainly due to novel scientific advances in understanding mechanisms involved in regulation of appetite, glucose, fat, and lipids metabolism. Examples of antiobesity drugs used nowadays in obesity management - grouped according to their actions - are: a. phentermine-topiramate (Qsymia^®^), setmelanotide (IMCIVREE^®^), naltrexone-bupropion (Contrave^®^), acting on the nervous system, b. orlistat (Xenical^®^) that inhibits lipase and thus fat absorption, c. liraglutide (Saxenda^®^), semaglutide (Wegovy^®^), which are glucagon like peptide 1 (GLP-1) receptor agonists, and tirzepatide (Mounjaro^®^) a dual glucose-dependent insulinotropic polypeptide, GIP, and GLP-1 receptor agonist (6, 7). The latter group of AOM is constantly drawing a substantial interest, due to the potent effects of these drugs, not only on weight loss and hyperglycemia, but also on other important cardiometabolic parameters such as dyslipidemia (1, 8).

Despite the progress in AOM, there are still major challenges. Side effects of these drugs remain an important issue as they can lead to discontinuation of therapy. Along with any contraindications, these are important factors limiting their use. Because of this, patients and clinicians may appear hesitant in using AOM. Moreover, cost and insurance coverage are additional matters of concern, not allowing routine use in clinical practice. Last, but not least, anti-obesity drugs are prescribed under certain clinical criteria, for example a BMI>30 or BMI>27 in presence of obesity-relative comorbidities, excluding the highly prevalent group of overweight. Surgical intervention can be indicated in cases and result in significantly higher weight loss compared to the drugs, but again the high cost and the related complications are restrictive factors (1, 7, 9–11).

In the need of effective, safe, and at the same time affordable and accessible interventions, research for natural anti-obesity compounds has increased importantly in the last decade. Natural products from plants and microorganisms have been used for centuries in therapeutics, while modern science constantly proves their pleiotropic value in health. Besides, even nowadays, pharmacopeia still develops and produces an important rate of drugs based on natural products (12).

*Morus alba* is a plant traditionally used for centuries in several health issues. Scientific research has revealed that it is a rich source of natural bioactive compounds, making it quite appealing for therapeutics. The scope of this paper is to provide comprehensive evidence (*in vitro*, *in vivo*, and clinical studies) on *Morus alba* effects in metabolic parameters associated with overweight and obesity and justify its potential valuable role in weight loss management.

## 2 Methods

A systematic electronic search of the literature was performed, from inception to the current date, using the following online databases: NCBI, Pubmed, and Google Scholar. The search strategy used a combination of keywords as follows: “Morus alba”, “Morus alba digestive enzymes”, “Morus alba glucose metabolism”, “Morus alba adiposity”, “1-DNJ glucose”, “Morus alba lipid metabolism”, “Morus alba metabolic syndrome”, “Morus alba antiobesity”.

The authors selected relevant studies initially by screening the abstracts. Articles that met the criteria were then further reviewed to determine whether they would be included in the present review. Inclusion criteria consisted of published articles, articles that were available in English, and included *in vitro*, *in vivo*, and clinical trials. Exclusion criteria consisted of duplicates, non-English articles, articles that did not report outcome measures, and papers that were unrelated to the scope of the review. Additional references were included after a review of the bibliography of the identified papers and review articles. Out of 300 citations, 127 relevant articles were selected and included in the present review.

## 3 Description and phytochemical components of *Morus alba*

*Morus alba*, also known as the white mulberry, belongs to the family of Moraceae. It is a small to medium-sized tree which grows rapidly. Noteworthy is its rapid release of pollen, which occurs at a speed greater than half the speed of sound (13). While the first cultivations of this plant were done for the development of earthworm 4700 years ago in China, it became widely known for its biological properties and is now cultivated in many regions across the world (e.g., America, Europe, Asia) (14). Notably, mulberry fruit has been designated as one of the first ethnomedicinal plants by the Chinese Ministry of Health in 1985 and was also recorded as a monograph in Chinese Pharmacopoeia (2015 edition). The various parts of this plant, such as the roots, stem, leaves, and fruits, are used for their various biological properties by many therapeutic methods such as ayurveda and traditional medicine. These properties are due to the abundant secondary metabolites (phytosterols, anthocyanins, sitosterols, flavonoids, morusimic acid, triterpenes, tannins, benzofuran derivatives, saponins, anthraquinones, glycosides etc.) contained in it and it is also rich in nutritional value as it contains proteins, carbohydrates, fiber, organic acids, vitamins, and minerals. **Table 1** presents several secondary metabolites of *Morus alba* which have been associated with various pharmacological properties.

**Table 1:** Main chemical components found in various parts of *Morus alba*.

Part of Morus alba	Chemical component	Reference
Fruits	caffeic acid	(14–18)
	atechinhydrate	
	chlorogenic acid	
	cyanidin 3-glucoside	
	cyanidin 3-rutinoside	
	cyanidin 3,5-diglucoside	
	gallic acid	
	epicatechin	
	coumaric acid	
	sinapic acid	
	salicylic acid	
	rutin	
	ellagic acid	
	myricitin	
	transcinamic acid	
	quercetin, quercetin 3-Orutinoside	
	quercetin 3-Oglucoside	
	quercetin 3-Orutinoside	
	quercetin 3-Ogalactoside	
	myricetin	
	kaempferol	
	kaempferol 3-O-glucoside	
	kaempferol 3-O-rutinoside	
	catechin	
	epigallocatechin gallate	
	epicatechin	
	procyanidin	
	pelargonidin 3-glucoside	
	cyanidin 3-O-(6′′ -O- α-rhamnopyranosyl-β-Dglucopyranoside)	
	cyanidin 3-O-(6′′ -O-arhamnopyranosyl-β-Dgalactopyranoside)	
	peonidin 3-glucoside	
	resveratrol	
	oxyresveratol	
	syringic acid	
	oxyresveratol (trans-2,31,4,51tetrahydroxystilbene-bioactives substances	
	vanilinic acid	
	cyanidin 3-O-β-Dgalactopyranoside	
	cyanidin 7-O-β-Dglucopyranoside	
	petunidin 3-O-β-glucopyranoside	
	chlorogenic acid	
	ferulic acid	
	p-Coumaric acid	
	0-Coumaric acid	
	cinnamic acid	
	protocatechuic acid	
	vanillic acid	
	mulberry fruit polysaccharides (FMAP, MFP, MFP-1, MFP-2, MP, PMF1,PMF2, PMF3, MFP3P, JS-MP-1)	
Leaves	1-Deoxynojirimycin (DNJ)	(19–21)
	rutin	
	quercetin-3-(6-malonyl) glucoside	
	isoquercitin	
	astragalin	
	quercetin 3-O-βglucopyranoside-7-O-α-rhamnopyranoside	
	kaempferol-7O-glucoside	
	quercetin-3-Orhamnopyranoside-7-Oglucopyranoside	
	oxyresveratol	
	5,7-dihydroxycoumarin 7-methyl ether	
Root bark	kuwanon S	(22–24)
	mulberroside A	
	mulberroside C	
	cyclomorusin	
	eudraflavone B hydroperoxide	
	oxydihydromorusin	
	leachianone G	
	alpha-acetyl-amyrin	
	moracins	
Wood	chlorogenic acid	(25)
	maclurin	
	oxyresveratol	

## 4 *Morus alba* in weight loss management: mechanisms of action

While *Morus alba* is well known for its antidiabetic effects, studies have revealed that, apart from lowering blood glucose levels, its bioactive compounds have a potential to yield significant advantages in additional metabolic processes involved in overweight and obesity. This section aims to present a summary of findings from both *in vitro* and animal studies, enlightening the primary mechanisms that make *Morus alba* an effective natural supplement in weight loss management.

### 4.1 Reduction of energy caloric intake via digestive enzymes inhibition

Caloric deficit is a primary requirement for weight loss. Regardless of how this will be achieved, the energy intake from diet should be less than the energy the body expends to perform even its basic metabolic functions. This negative energy balance should be at least 500 kcal/days to reach an effective weight reduction. At the same time, a balanced diet that ensures the necessary intake of protein and micronutrients needs to be ensured. A strategy of lowering the high energy substrates of carbohydrates and/or fat is a usual dietary pattern in weight loss programs. However, diets eliminating these groups of nutrients could result in lower adherence rates (at least in the long term), and micronutrients deficits. To enhance the desired caloric deficit, and at the same time support the adherence in the instructed dietary plan, one of the targets of the compounds used in weight management is the inhibition of carbohydrates or fat digestive enzymes. Inhibition of those enzymes can result in reduced absorption of dietary glucose and fats respectively and reduce the caloric energy intake (6, 26, 27).

Mulberry leaves extract has been extensively studied for its ability to inhibit carbohydrates digestion. These effects are mainly attributed to α-glucosidase inhibition. The IC50 values reported in studies vary, mainly depending on the extraction method. For example, Adisakwattan et al. (2012) reported IC50 values of 0.59 ± 0.06 and 0.94 ± 0.11 mg/ml on maltase and sucrase activities respectively, from an aqueous extract of Mulberry (28). Another study on an ethanolic *Morus alba* extract identified an IC50 value of 309.82 ± 5.72 μg/ml on α-glucosidase activity (29).

**Figure 1:**
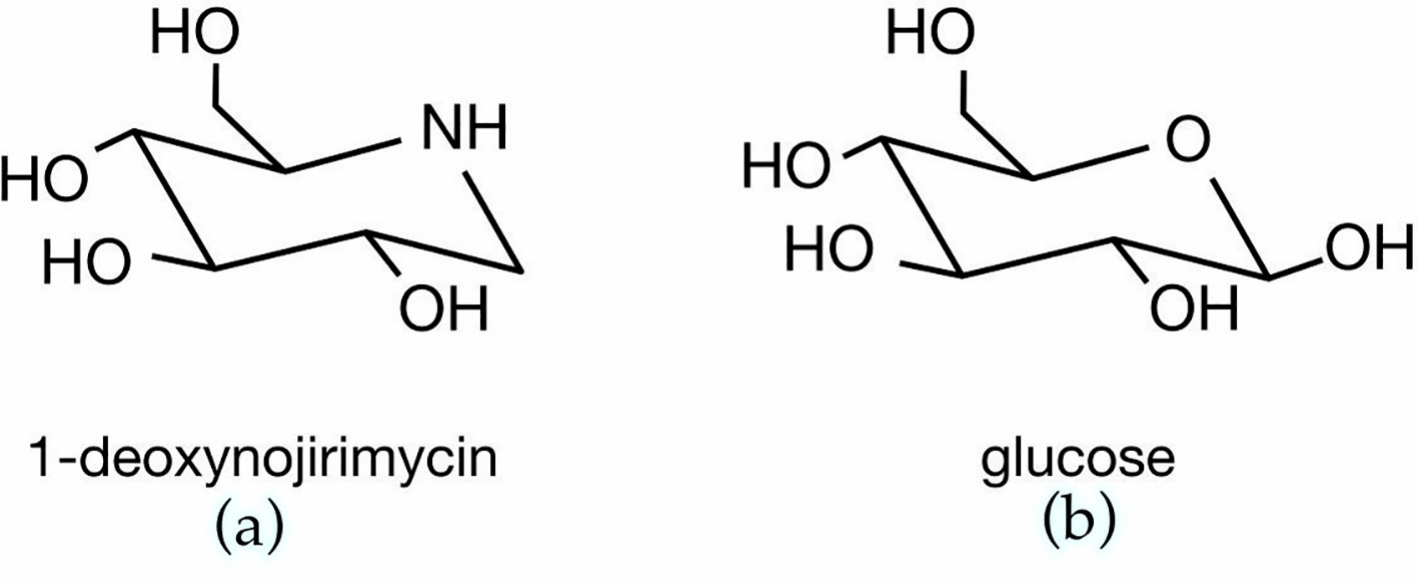
Chemical structure of **(A)** 1-deoxynojirimycin (1-DNJ) and **(B)** glucose.

*Morus alba’s* most well-known component, responsible for the inhibitory action on α-glucosidase, is 1-Deoxynojirimycin (1- DNJ). It has been shown that 1-DNJ, having a similar chemical structure to glucose (**Figure 1**), and a high binding affinity to the active site of α-glucosidase, inhibits in a competitive and reversible manner the digestive ability of this enzyme. It is noteworthy that 1-DNJ has shown equivalent or even superior α-glucosidase inhibitory activity to acarbose. Acarbose is a drug used for its glucose lowering effect in diabetes management, inhibiting both α-glucosidase and α-amylase, and it was also shown to result in weight loss. Wu et al. (2018) reported IC50 value of 1-DNJ on maltase activity 1.5 ± 0.1 × 10^-3^mol L^-1^ and for acarbose the relative value was 1.25 ± 0.05 × 10^-3^ mol L^-1^ (30), whereas an older study showed even higher differences in α-glucosidase inhibition, favoring 1-DNJ compared to acarbose (31).

With regards to pancreatic α-amylase, extracts from *Morus alba* have shown to possess an inhibitory activity to some extent, but this varied importantly based on the extraction method. Extraction of flavonoids from mulberry leaves with the organic extracts butanol and ethyl acetate, resulted in good inhibitory action, 66.67 μg/mL^-1^ and 88.00 μg/mL^-1^ respectively (32). However, *Morus alba’s* action on α-amylase was weaker than the inhibitory activity of acarbose. It should be noted that this lower activity may highlight Mulberry’s potential advantage over acarbose when it comes to possible adverse gastrointestinal side effects, a matter that will be reviewed in the further section about safety.

Furthermore, *Morus alba* extracts have demonstrated a pancreatic lipase activity, signifying their ability to reduce fat absorption from dietary intake and consequently reduce caloric intake from fat consumption. In a research study examining the impact of various edible plant extracts on lipase activity, Mulberry leaves exhibited the strongest effect, with an IC50 value of 0.01 ± 0.01 mg/mL. Nevertheless, the inhibitory activity of all plant extracts on lipase were less potent than that of orlistat (IC50 of 1.34 ± 0.13 μg/mL) (33) the anti-obesity medication inhibiting gastric and pancreatic lipases. Jeong et al. (2015) studied the inhibitory effects of distinct phenolic constituents from *Morus alba* leaves, determining that morachalcone A demonstrated significant activity at 6.2 μM (34). A relative study on phenolic compounds from root bark extracts of *Morus alba* showed potent inhibitory activities on lipase, ranging from 0.09 to 0.92 μM, compared to IC50 = 0.012 μM of orlistat, which was used as positive control (35).

### 4.2 Effects of *Morus alba* on glucose - insulin metabolism

Type 2 Diabetes is a chronic condition of hyperglycemia, defined by fasting blood glucose >126 mg/dL, or a 2-hour postprandial blood glucose >200 mg/dL. Overweight and obesity are closely related to insulin resistance, a disorder that precedes development of type 2 diabetes. The term “Diabesity” reflects this interlink between obesity and diabetes and is used by scientists to address - at least some of - their shared pathogenetic mechanisms (36, 37).

Despite the common belief that excessive weight gain is the primary event in type 2 diabetes, there is evidence showing that insulin resistance and obesity have a bidirectional association. That may explain - at least partially - the observational data showing that most patients with type 2 diabetes are obese, whereas overweight and obese diagnosed with type 2 diabetes seem to represent a minor fraction of total overweight/obese individuals (38–40). Furthermore, drugs that were initially designed for type 2 diabetes management (especially the latest ones, such as semaglutide, liraglutide, tirzepatide) resulted in important weight loss effects. These outcomes not only provided scientists with additional knowledge about the physiological roles of insulin in metabolism and body weight regulation, but also paved the way for the ongoing advances in anti-obesity medications. However, as noted earlier, these medications are associated with important side effects and are prescribed only under certain criteria (7, 11).

Insulin is a hormone produced by the β-pancreatic islets of Langerhans and has a central role in regulation of metabolism. Insulin is responsible for ensuring normal blood glucose levels facilitating cellular uptake of glucose and promoting glycogen synthesis in the liver, muscle, and adipose tissue. It is also involved in energy expenditure and appetite regulation. Insulin resistance refers to the disrupted glucose cellular uptake and subsequent glucose production by the liver (gluconeogenesis), leading to hyperglycemia. Hyperinsulinemia is the compensatory increase in insulin production and secretion from pancreas. Such disruptions may affect multiple features of the metabolic profile (lipid and protein metabolism, energy/caloric intake, body fat distribution) and functions of various tissues and organs (vessels, liver, pancreas, bones) (41).

*Morus alba* has been studied extensively for its antidiabetic properties. In this section we will review the basic molecular mechanisms of mulberry effects on glucose regulation (summarized in **Table 2**) supporting its role in management of crucial metabolic parameters for an effective and healthy weight loss.

**Table 2:** Molecular mechanisms of Morus alba effects on glucose metabolism.

Experimental model	Treatment with *Morus alba* (ML) extract/components, method of administration, duration of treatment	Molecular Mechanism	Reference
Improved Insulin Resistance			
Streptozotocin diabetic induced rats	Polysaccharide ML leaves extract, intragastrically, 200 mg/kg/day for 6 weeks	↑IRS-2/PI3K/Akt pathway	(42)
*db/db* mice	1-DNJ ML leaves extract intravenously 20, 40 and 80 mg/kg/day for 4 weeks	↑IRS1/PI3K/Akt pathway ↑GLUT4 translocation	(43)
Streptozotocin - induced diabetic rats	ML leaves extract, by oral gavage 2g/kg/day, for 4 weeks	↑IRS-1/PI3K/Akt pathway ↑GLUT4 expression	(44)
IR model of 3T3‐L1 adipocytes	Flavonoids ML leaves extract, 20 μg/mL	↑IRS-1/PI3K/Akt pathway	(45)
IR model of L6 skeletal muscle cells	Flavonoids ML leaves extract, 10μg/mL	↑AMPK phosphorylation ↑PGC-1α, GLUT4	(46)
*db/db* mice	Flavonoids ML leaves extract, orally, *1*80 mg/kg/day, 7 weeks	↑AMPK phosphorylation ↑PGC-1α, GLUT4 ↓ mitochondrial dysfunction	
*db/db* mice	ML leaves extract, by oral gavage 200 mg/kg/day, 500 mg/kg/day, 1-DNJ extract 40 mM/kg/day, for 35 days	↑IRS-1/PI3K/Akt pathway ↑GLUT4 expression	(47)
Reduced Glucose absorption			
Disaccharide inhibition test in rats	ML leaves extract, orally (ED50: 0.11g/kg for sucrose, 0.44g/kg for maltose, and 0.38g/kg for starch).	IC50: 3.2 μg/ml for sucrase, 10μg/ml for isomaltase, and 51 μg/ml for maltase.	(48)
Streptozotocin - induced diabetic mice	1-DNJ ML leaves extract intragastrically administered, 50 mg/kg/day	↓intestinal SGLT1, Na1/K1-ATP and GLUT2	(49)
	Deoxynojirimycin-polysaccharide mixture (DPM), 150 mg/kg/day for 90 days		
Reduced Oxidative Stress			
Streptozocin-diabetic rats	Dried mulberry leaf powder at 25% in the diet for 8 weeks	↑Hepatic antioxidants enzymes (G6PDH, GPx, GST, SOD)	(50)
Streptozotocin diabetic induced rats	Polysaccharide ML leaves extract, intragastrically, 200 mg/kg/day for 6 weeks	↓ (MDA) ↑SOD, CAT, GPx	(42)
High-sugar and high-fat diet rats	Polysaccharide ML extract 100 mg/kg/day by gavage	↑SOD, CCO, (SDH ↓MDA	(51)

AMPK, adenosine monophosphate activated protein kinase; CAT, catalase; CCO, cytochrome C oxidase; GLUT2, glucose transporter protein type 2; GLUT4, glucose transporter protein type 4; GPx, glutathione peroxidase; GST, glutathione transferase; G6PDH, dehydrogenase; IRS, insulin receptor substrate 1; MDA, malondialdehyde; Na^+^/K^+^-ATP, sodium–potassium adenosine triphosphatase; PGC-1α, peroxisome proliferator-activated receptor γ co-activator 1-α; PI3K, phosphatidylinositol-3-hydroxyl kinase; PIP3, phosphatidylinositol-3,4,5-trisphosphate; SGLT1, intestinal glucose transport proteins sodium/glucose cotransporter 1; SDH, succinate dehydrogenase; SOD, superoxide dismutase. The arrows ↓, ↑ refer to reduce and increase respectively.

#### 4.2.1 Insulin resistance

Research studies have shown that *Morus alba* components can improve insulin resistance through different molecular mechanisms.

**Figure 2:**
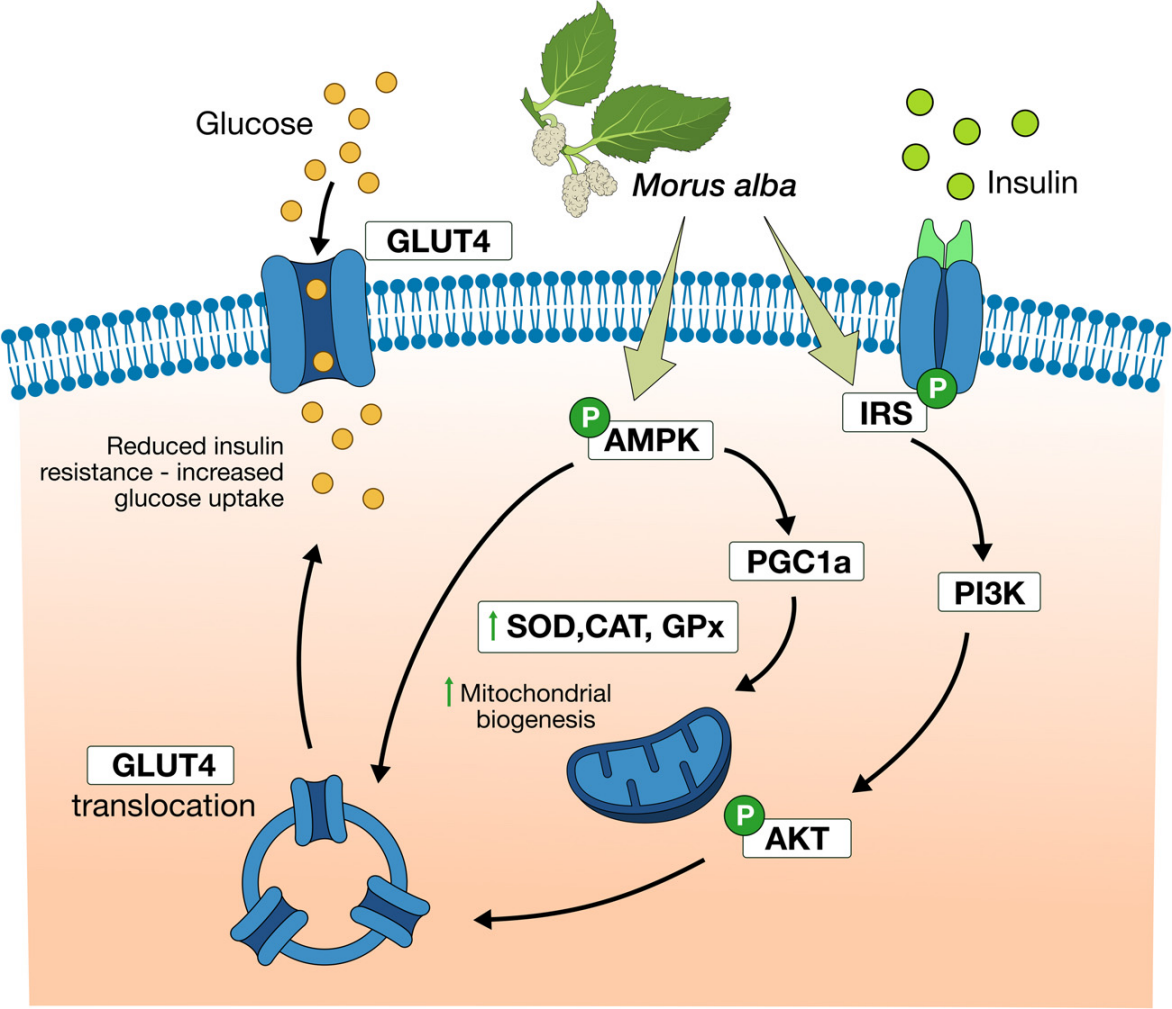
*Morus alba* effects in signaling pathways regulating glucose intake. Mulberry extracts activate IRS/PI3K/Akt pathway, increase GLUT4 expression and translocation to cellular membrane, thus improve glucose uptake. Moreover, Morus alba promote AMPK activation, mitochondrial biogenesis, and increase of antioxidant capacity.

The IRS/PI3K/Akt pathway is one of the most important signaling pathways in regulation of glucose metabolism in skeletal muscle, adipose, liver, and other tissues to promote glucose utilization by these tissues. Once insulin receptor substrate 1 (IRS-1) is activated, it stimulates phosphatidylinositol-3-hydroxyl kinase (PI3K), and synthesis of phosphatidylinositol-3,4,5-trisphosphate (PIP3), allowing phosphorylation of Akt, which results to glucose transporter protein type 4 (GLUT4) translocation to the cell membrane, thereby allowing glucose uptake in the target tissue (**Figure 2**). Disruptions in these signaling molecules are related to insulin resistance (41).

As shown in various experiments (*in vitro and in vivo)* Morus alba leaves extracts and its components (1-DNJ, flavonoids, polysaccharides) can improve glucose homeostasis via activation of the IRS-1/PI3K/Akt pathway and increased (glucose transporter type 4) GLUT4 expression and translocation. These experiments demonstrated mulberry’s effects in improvement of glucose tolerance, ameliorating insulin resistance, and in cases reducing body weight of animal models [41–46]. Moreover, Meng et al. (2020) showed that flavonoids from Morus alba extract promoted activation of adenosine monophosphate activated protein kinase (AMPK), another crucial signaling molecule in glucose homeostasis (46). AMPK activation increased peroxisome proliferator-activated receptor γ co-activator 1-α (PGC-1α), enhancing mitochondrial function, antioxidant capacity, GLUT4 expression, and translocation (**Table 2**, **Figure 2**).

#### 4.2.2 Postprandial glucose

Attenuation of postprandial hyperglycemia is another mechanism of *Morus alba* hypoglycemic effects. Normal blood glucose levels in response to meals are 140 mg/dL and typically return to premeal levels within 2-3 hours. Postprandial hyperglycemia is defined by blood glucose levels > 140 mg/dL, 1-2 hours after meal ingestion. Hyperglycemia after food intake is a risk factor for type 2 diabetes and cardiovascular disease (52).The inhibitory action of Morus alba components on carbohydrates digestive enzymes (α-glucosidase and α-amylase) has been repeatedly demonstrated (see paragraph 3) in several experiments. Due to this inhibitory action on digestion and absorption of carbohydrates, *Morus alba* extract can significantly reduce the postprandial glucose after meals intake that contain carbohydrates, as shown in animal models (48, 53) and in clinical studies, which will be reviewed in following paragraphs. Additionally, Li et al. (2013) showed that 1-DNJ can inhibit intestinal glucose absorption in the intestine by downregulation of the intestinal glucose transport proteins sodium/glucose cotransporter 1 (SGLT1), sodium–potassium adenosine triphosphatase (Na^+^/K^+^-ATP) and glucose transporter type 2 (GLUT2) expression (49) (**Table 2**).

#### 4.2.3 Oxidative stress

Mulberry extracts have shown to reduce oxidative stress in relation to diabetes. Oxidative stress refers to the imbalance between excessive generation of reactive oxygen (ROS) and nitrogen (NOS) species which the body’s endogenous antioxidants are inadequate to eliminate, leading to their accumulation with damaging effects on the cellular lipids, proteins, and DNA. Oxidative stress has been associated with disruptions in insulin signal transduction and harmful effects on pancreatic β-cells. Oxidative stress is also a pathogenetic mechanism linking obesity to insulin resistance: inflammatory adipokines released from excessive adipose tissue (especially visceral fat) and excessive free fatty acids result in systematic oxidation and insulin signaling disruption (54). Research studies have shown that mulberry leaves extracts can also ameliorate oxidative stress in diabetic mice, by enhancing cellular antioxidant enzymes, another beneficial effect on improving insulin resistance (**Table 2**).

#### 4.2.4 Gut microbiome

Latest research has demonstrated that *Morus alba* beneficial effects on glucose metabolism are also attributed to its actions on gut microbiota. In the past few years, an increasing number of studies have shown that the gut microbiome dysbiosis is related to type 2 diabetes and obesity. When the balance of the microorganism species that colonize the intestine is lost, the insulin signaling pathway and other metabolic functions may be disrupted. The concept of how gut microbiota affect the health of the host is quite recent and proves to be also importantly complicated. It has been proven though that intestinal microorganisms influence gut barrier function, immune response, and metabolism, via a range of their active metabolites (55).

Treatment of diabetic animal models with mulberry extracts seem to ameliorate such imbalances in gut microbiota. For example, Zhao et al. (2022), showed that polysaccharides from *Morus alba* leaf extract ameliorated metabolic disorders and reduced body, liver, and adipose tissue weight in obese mice. Investigating the effects of ML treatment on gut microbiome, they found that it increased gut microbiome diversity and reduced the abundance of *Mucispirillum*, *Dubosiella*, *Faecalibacullum*, *Lactococcus*, and *Desulfovibrio*, which were related to insulin resistance and adiposity indexes. On the other hand, ML extract increased the abundance of *Muribaculum*, *Bacteroides*, *Erysipelatoclostridium*, *Akkermansia*, and *Anaeroplasma*, which were correlated to metabolic disruptions (56). Similar effects of *Morus alba* components on relieving gut dysbiosis in relation to glucose homeostasis have been also observed in other studies (57–59).

## 5 Effects of *Morus alba* on lipid accumulation and adiposity

Obesity results from the excessive lipid accumulation in the body, leading to increased adipose tissue mass and disrupted regulation of lipid metabolism. Dyslipidemia is a metabolic disorder strongly associated with excessive body fat. In obesity, it usually presents with increased triglycerides (TG) and free fatty acids (FFA) and decreased high density lipoprotein-cholesterol (HDL-C) in plasma. However, low density lipoprotein (LDL-C) may be within normal range or slightly increased, but usually small dense LDL is elevated.

Insulin resistance, both in the adipose tissue and the liver, has been identified as a pathogenetic mechanism leading to disrupted lipids levels. Loss of insulin sensitivity results in increased release of FFAs in circulation from the hypertrophic adipocytes, increasing their uptake from the liver. Other sources of fatty acids in the liver are the *de novo* synthesis due to hyperinsulinemia and increased uptake of TG rich lipoproteins (chylomicrons) from plasma. Increased TG availability in the liver and increased secretion of very low-density lipoprotein (VLDL) from the liver into circulation, lead to enrichment of LDL and HDL particles with TG, and consequently accumulation of small, dense LDL and dysfunctional HDL (60–62).

Moreover, the enlarged and dysfunctional adipose tissue results in lipotoxicity, with increased markers of oxidative stress and low-grade inflammation, affecting metabolically active organs outside of the fat tissue, (liver, muscle, heart, brain) (63–65).

**Figure 3:**
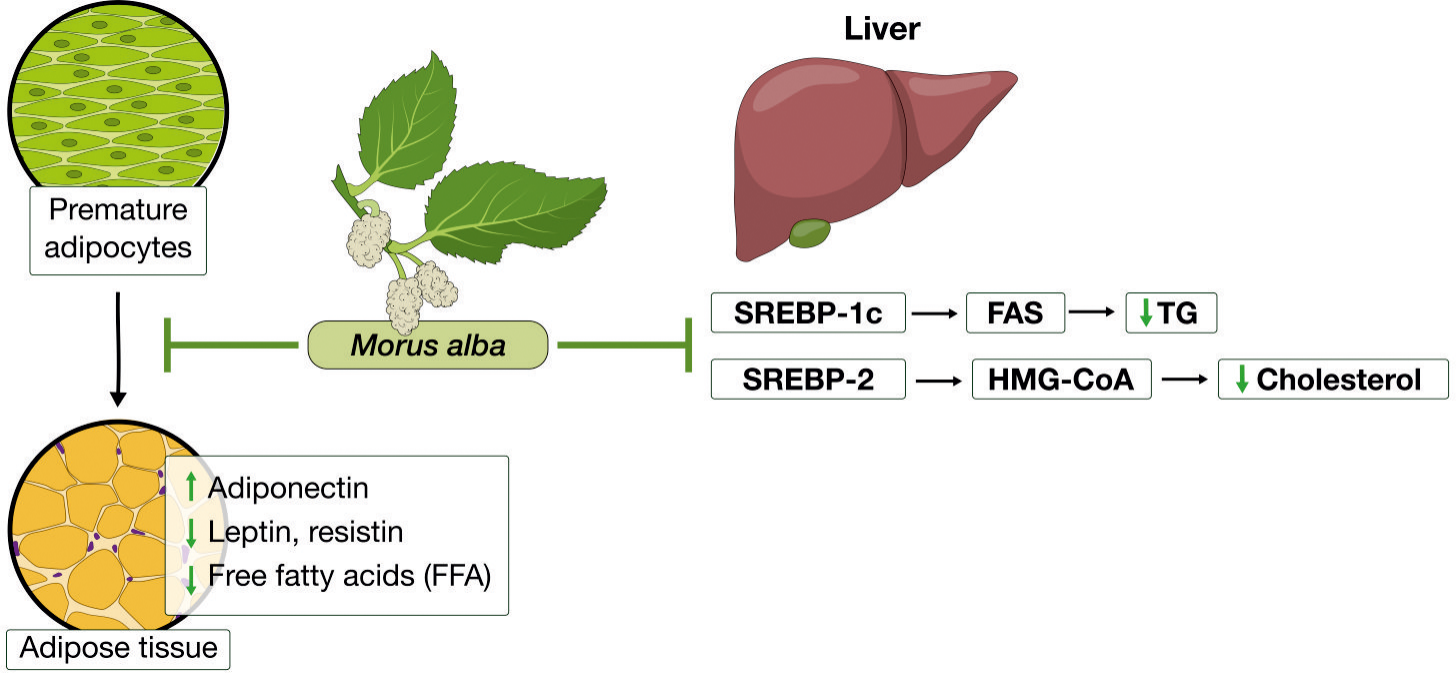
*Morus alba* effects in lipid metabolism and adiposity. In the adipose tissue, it inhibited adipogenesis, enhanced adiponectin levels, while reduced leptin, resistin and free fatty acids. In the liver, *Morus alba* reduced triglycerides and cholesterol, by inhibiting key mediators of their synthesis pathways.

*Morus alba* and its components have been found to improve importantly the disruptions of lipid accumulation and prevent body weight gain in obesity-induced animal models. Researchers investigating these effects have provided evidence on the molecular mechanisms by which *Morus alba* extracts affect lipid accumulation, dyslipidemia, and adipose tissue dysfunction which illustrated in summary in **Figure 3** and presented in detail in **Table 3**.

**Table 3:** Molecular mechanisms of mulberry leaves extracts in lipid metabolism and adiposity.

Experimental model	*Morus alba* extract or its components, dose, administration route, duration of treatment	Molecular Mechanism	Reference
Lipid metabolism regulation			
HepG2 cells	fruit extract, 1-6 mg/ml	↑ LDLR ↓ HMG-CoA ↓ SREBP-1c, FAS, GPAT	(66)
High fat diet-fed hamsters	fruit extract, 0.5, 1, 2% w/w, orally, 12 weeks	↓ HMG-CoA, FAS ↑ PPARα	(67)
High fat diet-fed mice	leaves extract, 1.5% w/w/day, orally, 6 weeks	↓ SREBP-1c,FAS ↓ SREBP-2, HMG-CoA ↑ AMPK phosphorylation	(68)
High cholesterol diet-fed mice	Polyphenol-rich leaves extract, orally, 0.1 and 1 mg/mL/day, 4 weeks	↓ HMG-CoA gene (*HMGCR)* expression) ↑ PPARα, PPARγ	(69)
HepG2 cells	leaves extract 3 mg/mL neochlorogenic acid (nCGA) 50, 150 μM	↓ SREBP-1c/FAS ↓ SREBP-2/HMG-CoA ↑ AMPK	(70)
Adipokines regulation			
High cholesterol diet-fed mice	leaves extract, 100 mg/kg/day, orally, 8 weeks	↓ Leptin, resistin ↑ Adiponectin	(71)
High fat diet-fed rat	fruit extract 150 mg/kg/day or 300 mg/kg/day, orally, 6 weeks	↓ Leptin level ↓ Leptin/adiponectin ratio ↓ PAI-1/adiponectin ratio	(72)
Oxidative Stress - Inflammation (obesity related) inhibition			
High fat diet-fed mice	Combination of leaves and fruit extract, 1:1 (200, 500, 1000 mg/kg/day) or 2:1 ratio (200, 500, 1000 mg/kg/day), stomach gavage, 12 weeks	↓ c-reactive protein (CRP), interleukin-1 (IL-1), (inducible nitric oxide iNOS synthase) ↓ NF-κβ ↑ HO-1, MnSOD	(73)
High fat diet-fed mice	leaves extract, 133 mg/kg/day and 666 mg/kg/day, orally, 12 weeks	↑ HO-1, GPx	(74)
HepG2 cells	leaves extract 3 mg/mL neochlorogenic acid (nCGA) 50, 150 μM	↓ NF-κB, TNF-α, IL-6	(75)
Adipogenesis inhibition			
3T3-L1 preadipocytes	Flavane and chalcone from leaves extract, 20,40, 80 μμ	↓ Differentiation of preadipocytes	(76)
3T3-L1 preadipocytes	leaves extract, 1 and 2 mg/mL, or polyphenols from leaves extract 0.25 and 0.5 mg/mL	↓ Differentiation of preadipocytes ↑ Adipocyte apoptosis (at highest doses)	(68)
3T3-L1 preadipocytes	Polysaccharide from fruit extract (JS-MP-1), 50, 100, 200, 500 μg/ml,	↑Cleaved caspase 9, cleaved caspase 3 and induced DNA fragmentation ↑ Adipocyte apoptosis	(77)

AMPK, adenosine monophosphate activated protein kinase; FAS, fatty acid synthase; GPAT, glycerol-3-phosphate acyltransferase; GPx, glutathione peroxidase; HMG-CoA, 3-Hydroxy-3-methylglutaryl coenzyme A reductase; HO-1, heme oxygenase; IL-6, interleukin 6; LDLR, low density lipoprotein receptor; MnSOD, manganese superoxide dismutase; NF-κB, nuclear factor kappa-B; PPAR-α, Peroxisome proliferator-activated receptors alpha; PPAR-γ, Peroxisome proliferator-activated receptors gamma; PAI-1, plasminogen activator inhibitor type 1; SREBP-1c, sterol regulatory element binding protein-1c; SREBP-2, sterol regulatory element binding protein 2. TNF-a, tumor necrosis factor alpha. The arrows ↓, ↑ refer to reduce and increase respectively.

### 5.1 Lipid metabolism

Important mediators of pathways regulating lipids synthesis which are disrupted in excessive fat accumulation are: sterol regulatory element binding protein-1c (SREBP-1c) that activates fatty acid synthase (FAS) and sterol regulatory element binding protein-1c (SREBP-2) which activates 3-Hydroxy-3-methylglutaryl coenzyme A reductase (HMG-CoA reductase), the rate-limiting enzyme in cholesterol biosynthesis. AMPK, apart from its role in glucose metabolism that we reviewed earlier, can also inhibit SREBs and thus regulate lipid metabolism (68, 75, 78). It has been shown that treatment with mulberry extracts can reduce total cholesterol as well as LDL and triglycerides and reduce fat accumulation. In cases there has been observed an increase in HDL. One of the key antilipidemic actions of mulberry lies in the expression of genes that regulate cholesterol and free fatty acids synthesis. For example, Liu et al. (2009) demonstrated that *Morus alba* fruit extract treatment in high cholesterol diet fed mice, reduced importantly TC and TG. Further *in vitro* experiments in HepG2 cells showed that this effect was due to an increased expression of low-density lipoprotein receptor (LDLR), whereas a downregulation in expression of HMG-CoA, SREBP1, FAS, glycerol-3-phosphate acyltransferase (GPAT) was shown (66). Similar effects were replicated in other studies too, proving mulberry’s beneficial effects in regulation of lipid accumulation (67–69, 75). We should also consider that TG increases in cases of a high carbohydrate intake. Thus, mulberry lowering effects on TG, could be a result, at least partially, of the 1-DNJ inhibitory effect on α-glucosidase, leading to inhibition of carbohydrates digestion (79) (**Table 3**).

### 5.2 Adipokines and inflammation

In addition to insulin resistance, adipokines produced from excessive fat tissue have an important role in dyslipidemia and obesity-related inflammatory states. Adipose tissue is known to act as an endocrine organ producing various types of adipokines, which take part in regulation of the metabolism and in inflammatory processes that develop in excessive adiposity. Leptin can regulate glucose and energy homeostasis and has similar effects with insulin on lipid metabolism. In obesity, similarly to insulin resistance, disruptions in leptin signaling occur, resulting in leptin resistance and high levels of leptin in plasma. Additionally, leptin acts as a pro-inflammatory adipokine, promoting low-grade inflammation. Resistin, another pro-inflammatory adipokine, has been associated with insulin resistance, mediating development of dyslipidemia. On the other hand, adiponectin is an anti-inflammatory adipokine, enhancing insulin sensitivity. Disruptions in adipokines levels due to hypertrophic fat cells have been associated with disruptions in pathways regulating lipid metabolism, leading to hypertriglyceridemia, increased FFA and reduced HDL (62, 80).

Apart from adipokines, excessive fat is associated with promotion of a general inflammatory state activating the major inflammatory pathway nuclear factor kappa-B (NF-κB) and consequently increase of inflammatory cytokines, such as tumor necrosis factor alpha (TNF-α) and interleukin 6 (IL-6). Oxidative stress, a known component of inflammatory state, is also increased, due to mitochondrial dysfunction and increased ROS production, in parallel with reduced antioxidative capacity (64, 65)

In recent studies, it was shown that *Morus alba* extract influences in a favorable manner the adipokines levels. Mulberry supplementation was capable of increasing adiponectin and reducing leptin in high fat fed mice (71, 72). With regards to inflammatory state and oxidative stress, mulberry has shown to reduce inflammatory markers related to obesity, such as TNF-a and IL-6 and decrease oxidative stress, by increasing antioxidant enzymes, such as heme oxygenase (HO-1), glutathione peroxidase (GPx) and manganese superoxide dismutase (MnSOD) (70, 73–75) (**Table 3**).

### 5.3 Adipogenesis

During a constant positive caloric intake that leads inevitably to weight gain, the adipose tissue stores excessive amounts of fat due to the excessive available fatty acids. This results in adipose hyperplasia, as the pre-adipocytes mature to new adipocytes, and consequently in increased fat tissue mass (hypertrophy). Relevant to the actions of *Morus alba* on lipid metabolism seem to be its anti-adipogenic effects observed in experiments studying hyperlipidemic models. Lim et al. (2013) showed that the combination treatment of leaves and fruit extracts in high fat fed obese mice, apart from promoting anti-hyperlipidemic effects, also reduced the epididymal adipocyte size (73). It was later confirmed in various *in vitro* experiments on 3T3-L1 adipocytes cells, that *Morus alba* and its components (flavone, chalcone and polysaccharides) were able to inhibit adipogenesis by inducing pre-adipocytes apoptosis and inhibiting their differentiation to mature adipocytes (68, 76, 77) (**Table 3**).

### 5.4 Gut microbiome

Anti-obesity and anti-hyperlipidemic effects of *Morus alba* extracts has been also attributed in latest studies to restoration of gut dysbiosis. In particular, Sojo et al. (2023), found that mulberry treatment in high-fat fed mice ameliorated the metabolic disruptions of lipid accumulation, prevented body weight gain and improved glucose tolerance. Researchers investigated the effects of the treatment on gut microbiota and found that *Morus alba* restored *Firmicutes/Bacteroidota* ratio and decreased plasma lipopolysaccharide (LPS) levels, indicative of obesity related inflammation (81). This beneficial effect of mulberry on mitigation of gut dysbiosis in relation to fat accumulation, is in accordance with a few earlier research studies (56, 81, 82). Interestingly, relevant results, attributing Morus antiobesity effects on gut microbiome changes, were also recently reported in dogs’ experiments. Researchers noticed a down regulation of gene expression CXCL8, whose increased expression is associated positively with obesity. Moreover, they reported increase of gut beta diversity and abundancy of the beneficial gut microbiome species *Lactobacillus ruminis* and *Weissella hellenica* (83).

## 6 Clinical studies

The aforementioned *in vitro* and *in vivo* studies provide strong evidence on the potential of *Morus alba* as an effective complementary compound in weight loss management. Its hypoglycemic properties are well established, and as we reviewed in the above paragraphs, there are important indications for its beneficial effects in regulating lipid metabolism and reducing fat accumulation. However, trials on humans are always essential to support its efficacy, along with its safety, in routine practice.

Till present, and to our knowledge, most clinical studies have focused on mulberry’s effects on glucose control. These trials (**Table 4**) were based on the 1-DNJ content of Morus alba extracts. As reviewed, 1-DNJ is mulberry’s component responsible to inhibit carbohydrates digestion. Therefore, most trials aimed to identify the effective dose that can result in a statistically significant postprandial glucose control after a single dose administration. Despite there is evidence for an effective dose of 1-DNJ being as low as 6 mg, we observed that most of the trials agree on the minimum of 12 mg 1-DNJ. These doses were standard for all participants, without differentiating the doses based on the participants’ body weight.

**Table 4:** Clinical studies demonstrating clinical efficacy of mulberry leaves extracts (MLE) on glucose/insulin metabolism.

Design of study	Subjects	Dose	Duration	Outcome	Reference
RCT	24 healthy	0.4, **0.8**, **1.2 g** MLE, 1.5% DNJ-enriched (6, **12, 18mg**)	Single dose	↓ Postprandial glucose and insulin reduced during sucrose tolerance test	(84)
Cross over, Double blind, RCT	12 with impaired FPG (100-140 mg/dL)	1.5% DNJ-enriched, (3, **6**, **9 mg** DNJ)	Single dose	↓ Postprandial glucose during high-carbohydrate tolerance test	(85)
	76 with impaired FPG (110–140 mg/dL)	1.5% DNJ-enriched, (6 mg DNJ), 3 times/day before meals	12 weeks	↑ 1,5-AG	
Double blind, RCT	50 healthy	1.25, **2.5** and **5 g**, 0.36% DNJ (4.5, **9**, **18** **mg** DNJ)	Single dose	↓ Postprandial glucose during maltose tolerance test	(86)
Double blind, RCT	36 with impaired FPG (100– 125 mg/dL)	5 g MLE, 0.36% DNJ **(18 mg)**, 3 times a day	4 weeks	↓ Postprandial glucose, insulin, C-peptide	(87)
Double blind, RCT	17 patients with T2D	1 g MLE, NA DNJ%, 3 times a day	12 weeks	↓ Postprandial glucose ↓Hemoglobin A1C (HbA1c) trend: from 7.30% to 6.94%, p = 0.079	(88)
Randomized, open-label, 7-cycle self-controlled crossover study	15 healthy	750 mg MLE, 1% DNJ	Single dose	↓ GIs for maltose (53.11%), sucrose (33.51%), maltodextrin (31.00%),glucose(8.12%)	(89)
Double blind, RCT	37 healthy	125, **250 mg**, **500 mg** MLE, 5% DNJ (6.75, **12.5**, **25 mg DNJ**)	Single dose	↓ Postprandial glucose and insulin during maltodextrin tolerance test	(90)
RCT	85 healthy	2.3, **4.6**, **6.9 g** MLE, 0.26% DNJ (6 mg, **12 mg**, **18 mg DNJ**)	Single dose	↓ Postprandial glucose during sucrose tolerance test	(91)
RCT	59 obese with borderline diabetes	**4.6 g** MLE, 0.26% DNJ **12 mg**, 3 times a day before meals	12 weeks	↓ FPG by 3.86 ± 5.99 mg/dL (p = 0.002) ↓ Hemoglobin A1C (HbA1c) by 0.11 ± 0.22% (p = 0.011)	
Double blind, RCT	38 healthy	250 mg MLE, 5% DNJ (12.5 mg DNJ)	Single dose	↓Postprandial glucose and insulin during sucrose tolerance test	(92)
RCT	30 healthy	250 mg MLE, 5% DNJ (12.5 mg DNJ)	Single dose	↓ Postprandial glucose response after a complete meal (Carbohydrates 57%, Sugars 4%, Protein 19%, Fat 24%)	(93)
RCT	37 healthy	200 mg, 225 mg, 250 mg MLE, 5% DNJ	Single dose	↓ Postprandial glucose and insulin response after a complete meal (Carbohydrate 60.9%, Protein 16.6%, Fat 20.4%)	(94)

When multiple doses have been tested in studies exploring the dose-effect relation, the dose associated with the mentioned outcome is indicated in bold. RCT, Randomized controlled study; 1.5-AG, 1,5-anhydroglucitol (a sensitive indicator of postprandial glycemic control); GI, Glycemic index; NA, not available, T2D, Type 2 Diabetes. The arrows ↓, ↑ refer to reduce and increase respectively.

It should be noted that 1-DNJ content in mulberry leaves is generally quite low, ranging of course among different cultivars from various geographical regions. Therefore, development of DNJ-enriched products is a strategy to enhance its absorption and efficacy. For example, a patented *Morus alba* leaves extract, standardized to contain 5% 1-DNJ was shown in four clinical studies to significantly reduce the glycemic response during either sucrose or maltodextrin tolerance test, or after a complete meal (containing all groups of macronutrients). Impressive reductions in post-glycemic response during sucrose tolerance test were observed: glucose iAUC reduced by 42%, (p = 0.001), insulin iAUC by 40%, (p < 0.001), peak glucose by 40.0%, (p < 0.001) and peak insulin by 41% (p < 0.001) from baseline (92). Recently, Thondre et al. (2024) investigated the effect of three different doses of this patented mulberry extract on blood glucose and insulin responses after eating a complex breakfast (providing a macronutrient composition of 60.9% carbohydrate, 16.6% protein and 20.4% fat). All three doses, 200 mg, 225 mg, 250 mg, significantly lowered glucose iAUC by 30% to (p = 0.003), 33% (p = 0.001) and 32% (p = 0.002), respectively, compared with placebo. All three doses significantly lowered the plasma insulin iAUC 120 by 31% (p = 0.024), 34% (p = 0.004) and 38% (p < 0.001), respectively (94).

Drawn from the previously mentioned preclinical data, *Morus alba* extracts can improve overall glucose metabolism, an effect attributed not only to 1-DNJ, but also to other constituents, such as flavonoids and polysaccharides. However, as one can review in **Table 4**, there is limited clinical evidence for the effects of mulberry extract intake on indexes such the fasting plasma glucose levels (FPG) or glycosylated hemoglobin (HbA1c). Riche et al. (2017), observed a trend in HbA1c reduction, which was not statistically significant (88), whereas Thaipitakwong et al. (2020), noticed an important reduction in both FPG and HbA1c in obese patients with borderline diabetes. After 12 weeks of MLE supplementation, the FPG was reduced by 3.86 ± 5.99 mg/dL (p = 0.002) and HbA1c by 0.11 ± 0.22% (p = 0.011) (91).

With regards to the meta-analyses which investigated the hypoglycemic effects of mulberry extracts, they all agree on its action of reducing importantly the postprandial glucose. However, the effects on fasting blood glucose and HbA1c were observed only in the most recent one by Cui et al. (2022) (32). It should be noted that all clinical trials so far seem to have important limitations. For example, the small number of participants, most of the trials performed on healthy individuals and, in cases, the absence of a placebo treatment group. Moreover, most of the clinical trials were based on a single-dose administration effect and the investigation of postprandial glucose response, rather than a study of long-term effects of *Morus alba* on glucose or other metabolic parameters.

It is noteworthy that, despite preclinical data about mulberry’s potent anti-hyperlipidemic effects, there is very scarce and weak clinical evidence available. In a small single group study in 10 subjects with hypertriglyceridemia, a modest reduction was observed after 12 weeks treatment with *Morus alba* extract (daily dose of 36 mg 1-DNJ for all participants regardless their body weight) (95). In another 12-week single group study the researchers noted a general improvement in subjects’ lipidemic profile: In patients with early-stage dyslipidemia daily supplementation of *Morus alba* leaves extract (280 mg/3 times a day before meals) reduced TG by 14.1%, (*p* < 0.05) and increased HDL-C by 19.7%, (*p* < 0.05) levels. It also reduced TC by 4.9%, (*p* < 0.05) and LDL-C by 5.6%, (*p* < 0.05) all compared to the baseline levels (96).

## 7 Safety profile of *Morus alba* extracts

To integrate the routine use of any compound in therapeutics - or in the management of health-related issues, such as body weight loss-, it is essential to acknowledge not only its efficacy but also its safety. With regards to *Morus alba* extracts, several toxicological data support its very good safety profile. Various experiments performed on animal models treated with *Morus alba* extracts provide evidence on quite high LD50 values and no-observed-adverse-effect level (NOAEL), compared to the dosages usually applied when studying mulberry’s beneficial pharmacological properties. There is, as expected, a variation in the values recorded in these studies, depending not only on the part of the plant investigated, but also on the method of extraction.

For example, oral administration of an ethanol extract from *Morus alba* leaves did not cause any acute genotoxicity in mice at doses of 75, 150 and 300 mg/kg for 14 days. Researchers observed that when the extract was given intraperitoneally, it did cause toxicity damage to the mice, which was importantly higher than the toxicity caused from oral administration (97). Chang et al. (2016) in 90 days repeated oral toxicity study, evaluated the sub chronic oral toxicity and genotoxicity of a water extract from *M. alba* fruits (MFE) in rats and reported a NOAEL greater than 1000 mg/kg, with no genotoxicity (98). Acute and subacute oral toxicity of *Morus alba* leaves water extract was also evaluated by Li et al. (2018). In this experiment LD50 was greater than 15 g/kg and NOAEL was 7.5 g/kg per day, the highest dose tested (99). A patented water extract from *Morus alba* leaves with 5% 1-DNJ, which is importantly higher than the usual 1.4 to 3.5 mg/g DNJ) was tested in a 28-Day repeated dose toxicological study. Researchers did not observe any treatment-related mortality and the NOAEL was 4000 mg/kg per day (100).

To our knowledge all clinical trials studying *Morus alba* effects in humans have shown that its intake is generally safe and well tolerated. Most usual side effects observed are gastrointestinal symptoms and no serious adverse events have been reported. In a meta-analysis study evaluating the glucose lowering effects of *Morus alba* the reported side effects included: nausea, loose stool, constipation, cramping, bloating, flatulence, distension, and proteinuria. The authors noted the absence of serious adverse events and that the side effects observed gradually disappeared over time (34).

The gastrointestinal side effects from *Morus alba* extracts intake are attributed to its 1-DNJ content. As reviewed earlier in this paper, 1-DNJ is a potent α-glucosidase inhibitor and -as proved from the effects on the postprandial glucose- can significantly reduce the absorption of glucose from carbohydrates. Although it can also attenuate pancreatic α-amylase activity, this inhibitory action is weaker. Acarbose on the other hand, strongly inhibits both enzymes. This results in the abnormal bacterial fermentation of the undigested complex carbohydrates in the large intestine that is most likely responsible for the significant gastrointestinal symptoms reported from acarbose intake. Therefore, *Morus alba* extracts could be considered as an effective but safer alternative to acarbose in reducing dietary carbohydrates absorption (28, 101)

Thaipitakwong et al. (2020) carried out a dose-finding study of mulberry 1-DNJ in non-diabetic, healthy persons based on its effects on postprandial glucose. Adverse events were monitored during the 180 min experiment and 7 days thereafter. Researchers considered a standard dose of 12 mg of DNJ as the optimal dose in terms of efficacy and safety, regardless of the participants body weight. They further conducted a long-term prospective study of 12 weeks in obese persons with borderline diabetes. Apart from the reported gastrointestinal side effects, no other life-threatening or severe complications were reported. The severity of the symptoms reported was acceptable by the participants of the study and the tolerance was improved over time (91). In three other clinical studies, single dose administration of the proprietary *Morus alba* leaves extract standardized to contain 5% DNJ (12.5 mg) was also in accordance with its previously reported well tolerability (90, 92, 93).

## 8 Discussion

Identification of effective weight loss strategies has become a paramount concern for health professionals worldwide. Scientists concur that, in addition to the “gold standard” of achieving a negative energy balance, losing weight should be a multidisciplinary approach. Lifestyle management comprises nutrition, exercise, and behavioral changes. A multimodal strategy can address more efficiently the substantial challenge of weight maintenance. However, it is noteworthy that despite losing weight can be successful utilizing a variety of approaches, most individuals will regain weight within 3 years. With regards to the dietary approach, it seems that in the long-term there is not an optimal diet that can guarantee more effective results. Thus, a dietary program that ensures intake of all essential nutrients, is tailored to individual needs, and considers personal preferences, is likely the most efficient. Adherence to any intervention program is considered the most important factor for a meaningful weight loss with long-term results. However, consistency to a healthy lifestyle may not be easy to achieve, since it can be undermined by an “obesogenic” environment: easy access to energy-dense foods that are rich in fat, sugar and salt, a sedentary job, and lack of time to exercise (6, 102–104).

Additional intervention with anti-obesity medications (AOM) may be required that can enhance the results from the lifestyle interventions. Despite the substantial progress in AOM development, there are still important factors limiting their routine use: they are recommended only for obese patients (or overweight with co-morbidities) and their side effects are considered important. Moreover, bariatric surgery, though very effective, is an option for severe cases. Apparently, there is a need for adjuvant approaches that can be both effective and safe in long-term use (1, 7, 10, 11).

**Figure 4:**
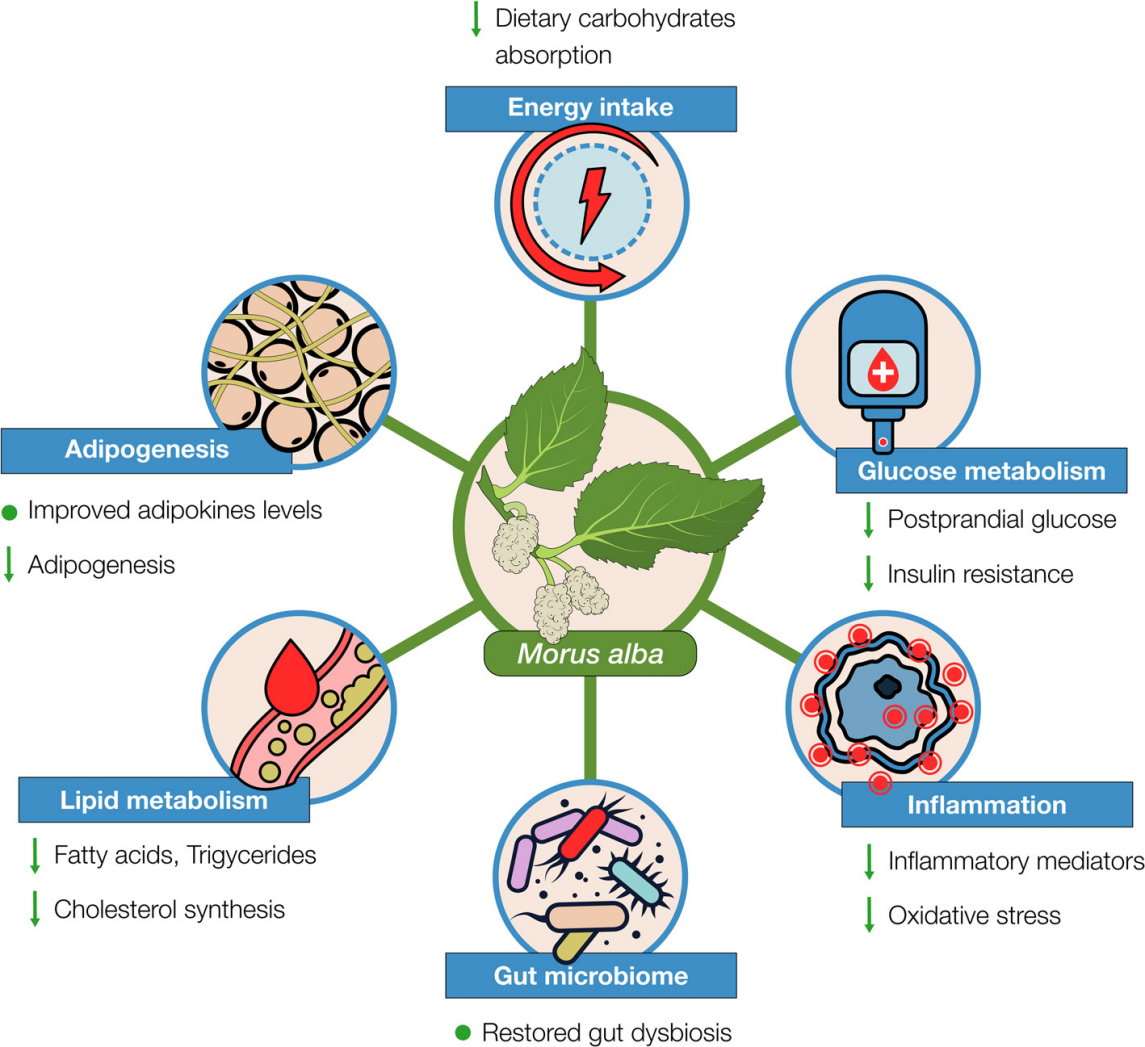
*Morus alba* effects on various aspects of weight loss management as evidenced from preclinical and clinical studies.

In this paper, we reviewed the evidence on *Morus alba* effects from preclinical and clinical studies that justify its use as a complementary treatment in weight loss management. From the data reviewed, Mulberry seems to provide a safe and pleiotropic support, not only in losing excessive weight, but also in regulation of important metabolic factors related to overweight and obesity (**Figure 4**).

With regards to the caloric deficit that is essential when losing weight, *Morus alba* can act beneficially by inhibiting importantly the absorption of dietary carbohydrates. As evidenced from the research, this effect is mainly attributed to its content in the alkaloid 1-DNJ inhibiting the digestive enzyme α-glucosidase (105). Moreover, mulberry extracts have shown to also possess - to some extent - an inhibitory effect on α-amylase. In relation to acarbose, *Morus alba* seems to have a comparable inhibitory effect on α-glucosidase, whereas for α-amylase, mulberry action is importantly weaker. This could be considered an advantage over acarbose’s notable gastrointestinal side effects, due to the important part of undigested complex carbohydrates and their fermentation by gut bacteria (28, 101). Mulberry has also shown inhibitory activity on lipase, though not comparable to that of orlistat (33, 35, 106). Overall, these inhibitory effects, particularly on α-glucosidase, could contribute importantly to reducing the caloric intake, especially from foods containing carbohydrates. It could also support dietary patterns that require important reductions of the carbohydrates’ intake (e.g., ketogenic), by allowing individuals to eat increased portions of carbs without affecting the desired impact of the diet. Additionally, *Morus alba* has shown to inhibit adipogenesis and regulate the endocrine effects of fat tissue, beneficially affecting levels of adipokines (leptin, adiponectin) (**Table 3**).

Additionally, *Morus alba* can -at least in preclinical studies- regulate glucose metabolism, attenuate insulin resistance, and improve lipidemic profile. It has been shown to increase the cellular glucose uptake, e.g., activating IRS-1/PI3K/Akt pathway and increase AMPK, and decrease expression of genes, such as SREBP-1, FAS, HMG-CoA resulting to reduction of lipids accumulation (**Table 2**). It is also noteworthy that there is some evidence on *Morus alba’s* positive effects in hypertension (107, 108) and atherosclerosis (22, 109). Metabolic syndrome, attributed to an important extent to overweight and obesity, is characterized by abdominal obesity, insulin resistance, hypertension, and hyperlipidemia. Inflammation, oxidative stress, and gut microbiome dysbiosis, are also counterparts of obesity and metabolic syndrome (110–112), in which mulberry - as we reviewed in this paper - has shown protective actions. Therefore, it can be suggested that mulberry’s beneficial effects are not limited to weight loss but can support overall metabolic and cardiovascular health.

It would be interesting for future research to investigate *Morus alba* potential actions in regulation of appetite and satiety. These effects could be achieved through different mechanisms: either directly on the brain nervous system or indirectly, through delayed digestion and ileal brake activation. Oh et al. (2009) investigated the effects of ethanol extract from Morus alba leaves on melanin-concentrating hormone (MCH) receptor that promotes food intake. Morus extract showed a potent inhibitory activity on MCH1 (113). In addition, Yimam et al. (2019) found that the flavonoids Kuwanon G and Albanin G in mulberry have a potent ligand binding inhibitory activity for cannabinoid receptor-1 (CB1). CB1 is involved in regulation of feeding behavior and is expressed in both brain and peripheral tissues. In their following *in-vivo* experiment, researchers showed that the *Morus alba* root bark extract enriched in Kuwanon G and Albanin G significantly reduced food intake both in short term (1 and 2 h post food) study. In the long term (7 weeks) treatment study with 500 mg/kg ML extract, apart from the reduction in food intake, researchers reported an important reduction in body weight, visceral fat deposit, and biochemical markers (fasting serum glucose, total cholesterol, and LDL-cholesterol in the obese induced animals. To our knowledge this is the only study about mulberry’s possible involvement feeding behavior through the nervous system (114).

Another possible way for mulberry’s action on appetite regulation may be related to the so-called “ileal brake”. This is a complex intestinal mechanism that may be triggered by undigested nutrients, such as whole grains that are rich in indigestible fibers. Undigested nutrients could promote satiety, either directly by delaying gastric emptying and reducing the passage rate of food), and/or via complex mechanisms through the nervous system, induced by hormones, such as the incretins (GIP and GLP-1) (115, 116). GIP and GLP-1 both promote insulin secretion after meal digestion and are also involved in regulation of food and energy intake. The understanding of incretins’ effects on insulin secretion has led recently to the development of antidiabetic drugs such as Ozempic^®^ (semaglutide, a GLP-1 agonist) and Mounjaro^®^ (tirzepatide, a double GLP-1 and GIP-1 agonist). Due to the important weight loss effects of these drugs, they are also used off-label in obesity management. Wegovy^®^, with enhanced dosage of semaglutide, was more recently developed and FDA-approved as an AOM (117). Moreover, the α-glucosidase inhibitor, acarbose, has been shown to promote GLP-1 production due to the delayed carbohydrates digestion (118). Since *Morus alba* extracts have shown potent inhibition of carbohydrates digestion and consequently glucose absorption, it would be interesting to explore its effects in activation of ileal brake, incretins, and thus regulation of satiety and food intake.

A useful strategy in development of nutraceutical products containing plant and natural compounds is to combine different extracts that act on similar biochemical pathways and thus may show synergic overall effects. This could also be the case for *Morus alba*, to enhance its efficacy in weight loss management. Yimam et al. (2017) tested a blend of extracts from *Morus alba, Yerba mate* and *Magnolia officinalis* for 7 weeks in different dosages, on a mouse model of obesity. They found that this plant extracts combination could importantly reduce body weight and improve glycemic and lipid profile. It is worth mentioning that the dose of 450 mg/kg of the plant extracts treatment reduced body fat distribution by 31.6%, whereas the reduction from treatment with orlistat was 17.2% (119). Kim et al. (2021) investigated the synergic effects of extracts from *Morus alba* and *Aronia melanocarpa* on an animal model for 14 weeks and this combination was also able to prevent weight gain and decrease liver lipid accumulation, adipocyte size in epididymal fat and abdominal fat volume (120). Combination of Morus alba with *Berberis aristata* and Monacolin on hepatocyte cell lines reduced PCSK9 expression, a key component for liver LDL receptors degradation, and thus resulted in increased LDL receptor and LDL uptake by the hepatocytes (121).

Based on the evidence reviewed in this paper, it is apparent that *Morus alba* has a strong potential as a supplementary approach in weight loss management. Among its major strengths as a nutraceutical, is its ability to address in a pleiotropic manner several aspects of weight regulation. Not only it can contribute to reduced energy intake when consumed with meals containing carbohydrates but can also help in regulation of crucial metabolic comorbidities in overweight and obesity, such as insulin resistance and dyslipidemia. In addition, with regards to its safety profile, *Morus alba* shall be considered safe and generally tolerable, with minor adverse effects, mainly gastrointestinal symptoms, that most likely dissolve in the long-term use.

However, with regards to the efficacy of *Morus alba* treatment in humans, we acknowledged that there are specific limitations. The clinical trials were focused on its antihyperglycemic effects, and particularly on reducing the levels of postprandial glucose. Thus, short-term, or single doses administration is not adequate to evaluate the clinical efficacy of *Morus alba* on regulation of glucose. Moreover, the number of participants in most clinical studies were relatively low and performed mostly in healthy individuals. As for *Morus alba* antihyperlipidemic effects, there is a lack of clinical evidence on its ability to improve the disrupted lipid profile in dyslipidemic patients, such as reducing the levels of TC, LDL or TG. Moreover, we could not retrieve any study evaluating its properties in weight loss management in humans. Therefore, we recognize the crucial need for the conduction of clinical trials that will be appropriately designed and performed on a long-term basis (at least 12 weeks) in overweight and/or obese individuals, evaluating *Morus alba* properties in body weight, and in metabolic parameters such as glucose and lipids regulation. We shall note however, that despite the lack of human studies evaluating *Morus alba* in humans with regards to its efficacy in weight loss and relative metabolic parameters, there is clinical evidence for some of its major components’ effects. For example, chlorogenic acid is implicated in reduction of body weight, as well as improvements in glucose and lipids levels (122–124). Moreover, several flavonoids (resveratrol, epigallocatechin gallate, quercetin, and others) are in the research interest because of their ability to beneficially modulate various cardiometabolic parameters as shown in clinical trials, such as the lipid profile, blood pressure, and blood glucose (125–127).

## 9 Conclusion

There is an undoubted need for adjuvant options in weight loss treatments that can be both safe and effective for routine use, addressing a wider range of overweight and obese cases. Based on the evidence reviewed in this paper, *Morus alba* extracts could justify such a role in healthy weight management. More extended clinical trials are required to provide stronger evidence on white mulberry’s overall beneficial effects in weight reduction and metabolic disruptions in humans.
